# Complete Genome Sequence of *Phaeobacter* sp. Strain G2, Isolated from a Lab Culture of the Calcifying Alga Emiliania huxleyi

**DOI:** 10.1128/mra.01067-22

**Published:** 2022-11-30

**Authors:** Siwat Chang, Tzu-Haw Wang, Ming-Wei Lai, Chuan Ku

**Affiliations:** a Institute of Plant and Microbial Biology, Academia Sinica, Taipei, Taiwan; University of Southern California

## Abstract

We report the complete genome sequence of *Phaeobacter* sp. strain G2, which was isolated from a lab culture of the coccolithophore alga Emiliania huxleyi strain RCC 1216. The 4,963,472-bp sequence has a G+C content of 58.85% and was assembled using Illumina short reads and Oxford Nanopore Technologies long reads.

## ANNOUNCEMENT

Alga-bacterium interactions are important factors in the metabolism, physiology, and ecology of microbial communities in aquatic ecosystems (1). Emiliania huxleyi is a globally distributed microalga with a complex evolutionary history (2), best known for its ability to calcify and form massive algal blooms that have major impacts on the biogeochemical cycling of carbon and other elements (3). Notable examples of its interactions with bacteria include the algicidal effects by a *Sulfitobacter* strain (4, 5) and a *Phaeobacter* strain (6), both of which belong to the order *Rhodobacterales* (*Alphaproteobacteria*). How the interactions with *E*. *huxleyi* vary across closely related bacteria with different gene contents is poorly understood. Here, we report the complete genome sequence of a nonalgicidal *Phaeobacter* strain in association with cultured *E*. *huxleyi*.

*Phaeobacter* sp. strain G2 was isolated from a lab culture of the calcifying *E. huxleyi* strain RCC 1216 (K/2 medium [7] at 18°C with a light/dark cycle of 16 h/8 h) in January 2021. The culture was filtered through a 3-μm polycarbonate filter, and the filtrate was then plated onto a 1% agar plate prepared from K/2 medium supplemented with 1% yeast extract and 5% Bacto Peptone (Becton, Dickinson). A white-pinkish colony was picked and cultured in liquid medium at 18°C. Genomic DNA was extracted using the Quick-DNA fungal/bacterial miniprep kit (Zymo Research) according to the manufacturer’s instructions. An Illumina sequencing library was prepared using the Nextera XT DNA library preparation kit (Illumina) and sequenced on a NovaSeq 6000 instrument, producing a total of 57,144,203 150-bp paired-end reads. Another DNA sample for Oxford Nanopore Technologies (ONT) sequencing was prepared from a separate inoculum. The ONT sequencing library was prepared without size selection using the ligation sequencing kit SQK-LSK109 with native barcoding (ONT) and was sequenced on the MinION Mk1C system with an R9.4.1 flow cell (ONT). Read base calling was performed using Guppy v. 6.0.7, which is an accurate base-caller for ONT data (8). This generated 627,900 reads with an *N*_50_ value of 12.76 kbp. Filtlong v. 0.2.1 (9) was used to filter out reads shorter than 1 kbp and 5% of reads that were of low quality. We *de novo* assembled the *Phaeobacter* sp. strain G2 genome sequence with both the Illumina and ONT reads using the Trycycler v. 0.5.3 (10) pipeline with default parameters. The Illumina reads were first quality filtered using fastp v. 0.22.0 (11). Flye v. 2.9-b1768 (12), Raven v. 1.8.1 (13), and Miniasm/Minipolish v. 0.1.3 (14) were used to generate 12 input assemblies for Trycycler, which was used to reconcile these assemblies, generate a consensus assembly, and circularize the replicons. The consensus assembly was further polished with the filtered Illumina reads according to the Trycycler user manual using Polypolish v. 0.5.0 (15) and POLCA v. 4.0.5 (16). Default parameters were used for all software unless otherwise specified.

The complete genome assembly has a G+C content of 58.85% and contains 7 circular replicons with a total length of 4,963,472 bp. The replicons include 1 chromosome of 4,141,732 bp and 6 plasmids that were determined based on their small size, ranging from 199,094 to 105,595 bp. The NCBI Prokaryotic Genome Annotation Pipeline (PGAP) v. 6.2 (17) was used for functional gene annotations and predicted 4,510 protein-coding genes, 15 rRNAs, and 59 tRNAs.

### Data availability.

The *Phaeobacter* sp. strain G2 genome sequence has been deposited at GenBank under the accession numbers CP104100 (chromosome) and CP104101 to CP104106 (plasmids). The version described in this paper is the first version. The GenBank assembly accession number is GCA_025163595.1, the BioSample accession number is SAMN30679372, and the BioProject accession number is PRJNA877062. The Illumina and Oxford Nanopore raw reads are available in the Sequence Read Archive under the accession numbers SRR21747910 and SRR21747909, respectively.
